# Bis(4-amino-3,5-di-2-pyridyl-1,2,4-triazole-κ^2^ *N* ^1^,*N* ^5^)diaqua­zinc(II) dinitrate

**DOI:** 10.1107/S1600536809032103

**Published:** 2009-08-22

**Authors:** Jia Hua, Lu Gao, Baiyan Li

**Affiliations:** a State Key Laboratory of Inorganic Synthesis and Preparative Chemistry, College of Chemistry, Jilin University, Changchun 130012, People’s Republic of China

## Abstract

The asymmetric unit of the title compound, [Zn(C_12_H_10_N_6_)_2_(H_2_O)_2_](NO_3_)_2_, contains one-half of the complex molecule and one NO_3_^−^ anion. The Zn^II^ ion displays a distorted tetra­gonal-pyramidal geometry with four N atoms from two chelating 4-amino-3,5-di-2-pyridyl-1,2,4-triazole (2-bpt) lig­ands in the basal plane and one water mol­ecule occupying the apical site. Another water mol­ecule at the opposite of the apical site has a weak inter­action with the Zn^II^ ion [Zn—O = 2.852 (5) Å]. The Zn^II^ ion and the two water mol­ecules lie on a twofold rotation axis. An extensive system of hydrogen bonds involving the NH_2_ groups of the 2-bpt ligands, water mol­ecules and nitrate anions links all residues into a three-dimensional network.

## Related literature

For transition metal complexes of 4-amino-3,5-di-2-pyridyl-1,2,4-triazole (2-bpt), see: Shao & Geng (2009 ▶); Hartmann & Vahrenkamp (1995 ▶); Keij *et al.* (1984 ▶); Kitchen *et al.* (2008 ▶); Koningsbruggen *et al.* (1998 ▶); Tong *et al.* (2007 ▶). For rare earth metal complexes of 2-bpt, see: Garcia *et al.* (1986 ▶); Rheingold *et al.* (1993 ▶). For hydrogen-bonding inter­actions involving 2-bpt in organic compounds, see: Mernari *et al.* (1998 ▶).
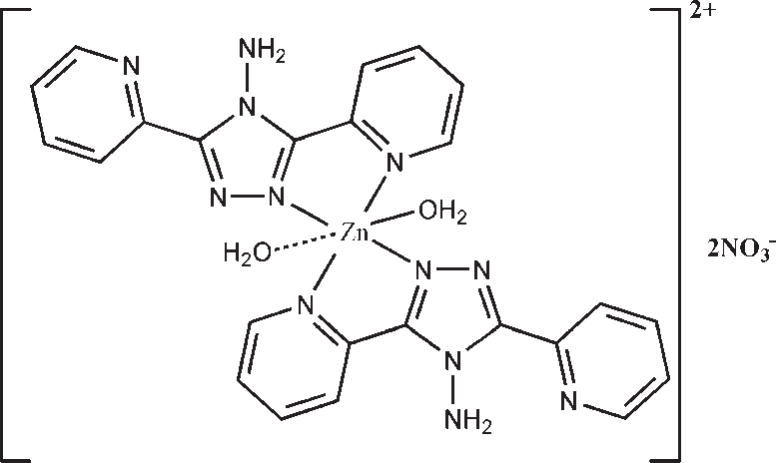


## Experimental

### 

#### Crystal data

[Zn(C_12_H_10_N_6_)_2_(H_2_O)_2_](NO_3_)_2_
*M* *_r_* = 701.94
Monoclinic, 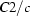

*a* = 14.856 (3) Å
*b* = 9.4185 (19) Å
*c* = 20.230 (4) Å
β = 91.99 (3)°
*V* = 2829 (1) Å^3^
*Z* = 4
Mo *K*α radiation
μ = 0.95 mm^−1^
*T* = 298 K
0.25 × 0.20 × 0.20 mm

#### Data collection

Bruker SMART 1000 CCD diffractometer
Absorption correction: multi-scan (*SADABS*; Sheldrick, 1996 ▶) *T*_min_ = 0.797, *T*_max_ = 0.828
13550 measured reflections
3210 independent reflections
2424 reflections with *I* > 2σ(*I*)
*R*_int_ = 0.067

#### Refinement

*R*[*F* ^2^ > 2σ(*F* ^2^)] = 0.045
*wR*(*F* ^2^) = 0.101
*S* = 1.03
3210 reflections
228 parameters
H atoms treated by a mixture of independent and constrained refinement
Δρ_max_ = 0.32 e Å^−3^
Δρ_min_ = −0.64 e Å^−3^

### 

Data collection: *SMART* (Bruker, 2007 ▶); cell refinement: *SAINT* (Bruker, 2007 ▶); data reduction: *SAINT*; program(s) used to solve structure: *SHELXS97* (Sheldrick, 2008 ▶); program(s) used to refine structure: *SHELXL97* (Sheldrick, 2008 ▶); molecular graphics: *DIAMOND* (Brandenburg, 1999 ▶) and *SHELXTL* (Sheldrick, 2008 ▶); software used to prepare material for publication: *SHELXTL*.

## Supplementary Material

Crystal structure: contains datablocks global, I. DOI: 10.1107/S1600536809032103/hy2215sup1.cif

Structure factors: contains datablocks I. DOI: 10.1107/S1600536809032103/hy2215Isup2.hkl

Additional supplementary materials:  crystallographic information; 3D view; checkCIF report

## Figures and Tables

**Table 1:** Selected bond lengths (Å)

Zn1—O1*W*	2.008 (3)
Zn1—O2*W*	2.852 (5)
Zn1—N1	2.078 (2)
Zn1—N5	2.141 (2)

**Table 2:** Hydrogen-bond geometry (Å, °)

*D*—H⋯*A*	*D*—H	H⋯*A*	*D*⋯*A*	*D*—H⋯*A*
N4—H4*A*⋯O2^i^	0.94 (4)	2.25 (4)	3.100 (3)	149 (3)
N4—H4*A*⋯O2	0.94 (4)	2.55 (4)	3.192 (4)	126 (3)
N4—H4*B*⋯N6	0.89 (4)	2.13 (4)	2.875 (3)	141 (3)
N4—H4*B*⋯O3	0.89 (4)	2.43 (4)	3.005 (3)	122 (3)
O1*W*—H1*W*⋯O3^ii^	0.79 (3)	1.93 (3)	2.714 (3)	170 (3)
O2*W*—H2*W*⋯O1^iii^	0.92 (5)	2.02 (5)	2.900 (4)	161 (5)

Symmetry codes: (i) 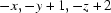; (ii) 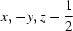; (iii) 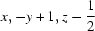.

**Fig. 1.**
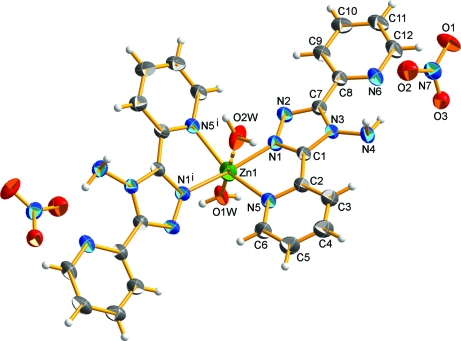
Molecular structure of the title compound. Displacement ellipsoids are drawn at the 50% probability level. [Symmetry code: (i) -x, y, 3/2 - z.]

**Fig. 2.**
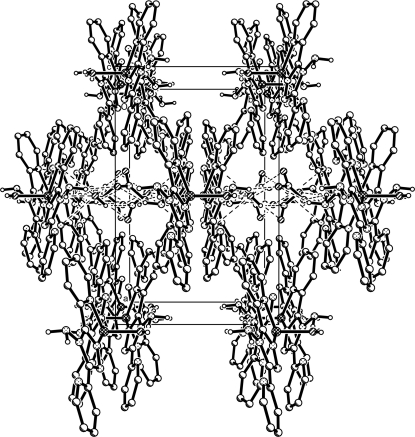
The crystal packing of the title compound. Dashed lines denote hydrogen bonds.

## supplementary crystallographic information

### Comment

Recently, 4-amino-3,5-di-2-pyridyl-1,2,4-triazole (2-bpt) has been used as ligand because it has potential ability of multi-coordination modes, generating hydrogen-bonding and/or aromatic stacking interactions. The zinc(II) complex of 2-bpt has been reported previously. Hartmann & Vahrenkamp (1995) prepared the complexes based on 2-bpt with Zn(ClO_4_)_2_, ZnCl_2_, Zn(NO_3_)_2_ and Zn(BF_4_)_2_. But they just obtained the single-crystal structure of Zn(ClO_4_)_2_ complex, in which Zn^II^ atom adopts octahedral geometry. Herein, we reported the crystal structure of the title compound obtained by reacting 2-bpt with Zn(NO_3_)_2_, in which 2-bpt acts as a chelating ligand and hydrogen-bonding interactions play an important role in stabilizing the solid-state structure.

The structure analysis reveals that the asymmetric unit of the title compund contains half a Zn^II^ atom, one 2-bpt molecule, two half water molecules and one NO_3_^-^ anion. As depicted in Fig. 1, the distorted tetragonal pyramidal Zn^II^ atom is located on an inversion center. The basal plane is defined by four N atoms from two 2-bpt ligands with a chelating mode, and the apical site is occupied by one water molecule (O1W) with a Zn—O distance of 2.008 (3)Å (Table 1). Another water molecule (O2W) has a weak interaction with the Zn atom [Zn1—O2W = 2.852 (5) Å], which makes the crystal structure differ from the recently reported nickel complex (Shao & Geng, 2009) because of Jahn-Teller effect. The 2-bpt molecule exhibits *trans*-conformation. The two 2-bpt molecules are not coplanar, with a dihedral angle of 48.54 (5)°. N—H···O, N—H···N and O—H···O hydrogen bonds are observed in the crystal structure (Table 2; Fig. 2).

### Experimental

To an ethanol solution (20 ml) of 2-bpt (0.012 g, 0.05 mmol) was added an aqueous solution (5 ml) of Zn(NO_3_)_2_.6H_2_O (0.018 g, 0.05 mmol) with stirring. After vigorous stirring for *ca* 40 min, the resultant solution was filtered and left to stand at room temperature. Colorless block crystals suitable for X-ray analysis were produced by slow evaporation of the solvent for two weeks.

### Refinement

C-bound H atoms were positioned geometrically and allowed to ride on their parent atoms, with C—H = 0.93 Å and with *U*_iso_(H) = 1.2*U*_eq_(C). H atoms attached to N atom were found in a difference Fourier map. Their coordinates were refined and *U*_iso_ factors were fixed. H atoms of water molecules were found in a difference Fourier map and refined isotropically.

### Crystal data

**Table:**

[Zn(C_12_H_10_N_6_)_2_(H_2_O)_2_](NO_3_)_2_	*F*(000) = 1440
*M**_r_* = 701.94	*D*_x_ = 1.648 Mg m^−^^3^
Monoclinic, *C*2/*c*	Mo *K*α radiation, λ = 0.71073 Å
Hall symbol: -C 2yc	Cell parameters from 3210 reflections
*a* = 14.856 (3) Å	θ = 3.2–27.4°
*b* = 9.4185 (19) Å	µ = 0.95 mm^−^^1^
*c* = 20.230 (4) Å	*T* = 298 K
β = 91.99 (3)°	Block, colorless
*V* = 2829 (1) Å^3^	0.25 × 0.20 × 0.20 mm
*Z* = 4	

### Data collection

**Table:**

Bruker SMART 1000 CCD diffractometer	3210 independent reflections
Radiation source: sealed tube	2424 reflections with *I* > 2σ(*I*)
graphite	*R*_int_ = 0.067
φ and ω scans	θ_max_ = 27.4°, θ_min_ = 3.2°
Absorption correction: multi-scan (*SADABS*; Sheldrick, 1996)	*h* = −19→19
*T*_min_ = 0.797, *T*_max_ = 0.828	*k* = −12→12
13550 measured reflections	*l* = −22→26

### Refinement

**Table:**

Refinement on *F*^2^	Primary atom site location: structure-invariant direct methods
Least-squares matrix: full	Secondary atom site location: difference Fourier map
*R*[*F*^2^ > 2σ(*F*^2^)] = 0.045	Hydrogen site location: inferred from neighbouring sites
*wR*(*F*^2^) = 0.101	H atoms treated by a mixture of independent and constrained refinement
*S* = 1.03	*w* = 1/[σ^2^(*F*_o_^2^) + (0.0336*P*)^2^ + 4.1918*P*] where *P* = (*F*_o_^2^ + 2*F*_c_^2^)/3
3210 reflections	(Δ/σ)_max_ < 0.001
228 parameters	Δρ_max_ = 0.32 e Å^−^^3^
0 restraints	Δρ_min_ = −0.64 e Å^−^^3^

### Fractional atomic coordinates and isotropic or equivalent isotropic displacement parameters (Å^2^)

**Table:**

	*x*	*y*	*z*	*U*_iso_*/*U*_eq_	
Zn1	0.0000	0.04107 (5)	0.7500	0.03188 (15)	
O1W	0.0000	−0.1721 (3)	0.7500	0.0441 (8)	
O2W	0.0000	0.3439 (5)	0.7500	0.1050 (19)	
O1	0.09893 (16)	0.4526 (3)	1.17312 (12)	0.0646 (7)	
O2	0.03688 (16)	0.4946 (2)	1.07668 (12)	0.0586 (6)	
O3	−0.01516 (15)	0.3294 (2)	1.13778 (10)	0.0511 (6)	
N1	0.03975 (14)	0.0690 (2)	0.84865 (10)	0.0298 (5)	
N2	0.11637 (14)	0.0466 (2)	0.88711 (10)	0.0304 (5)	
N3	0.01446 (13)	0.1534 (2)	0.94627 (9)	0.0250 (4)	
N4	−0.03229 (16)	0.2187 (3)	0.99862 (11)	0.0345 (5)	
N5	−0.12105 (14)	0.1149 (2)	0.79303 (10)	0.0305 (5)	
N6	0.13761 (15)	0.1407 (2)	1.06063 (10)	0.0335 (5)	
N7	0.04037 (16)	0.4274 (2)	1.12933 (12)	0.0371 (5)	
C1	−0.02138 (16)	0.1310 (3)	0.88464 (11)	0.0251 (5)	
C2	−0.11171 (16)	0.1616 (3)	0.85638 (12)	0.0267 (5)	
C3	−0.18075 (18)	0.2271 (3)	0.88886 (14)	0.0349 (6)	
H3	−0.1720	0.2599	0.9320	0.080*	
C4	−0.26341 (18)	0.2426 (3)	0.85559 (15)	0.0409 (7)	
H4	−0.3112	0.2859	0.8762	0.080*	
C5	−0.27402 (18)	0.1930 (3)	0.79131 (15)	0.0428 (7)	
H5	−0.3291	0.2014	0.7684	0.080*	
C6	−0.20152 (18)	0.1311 (3)	0.76189 (13)	0.0378 (7)	
H6	−0.2087	0.0990	0.7185	0.080*	
C7	0.10017 (17)	0.0992 (3)	0.94616 (12)	0.0268 (5)	
C8	0.16653 (17)	0.0977 (3)	1.00174 (12)	0.0282 (5)	
C9	0.25491 (18)	0.0556 (3)	0.99173 (13)	0.0331 (6)	
H9	0.2726	0.0256	0.9503	0.080*	
C10	0.31574 (18)	0.0597 (3)	1.04516 (15)	0.0393 (7)	
H10	0.3750	0.0306	1.0404	0.080*	
C11	0.2877 (2)	0.1073 (3)	1.10541 (15)	0.0416 (7)	
H11	0.3279	0.1131	1.1415	0.080*	
C12	0.1988 (2)	0.1460 (3)	1.11125 (13)	0.0381 (6)	
H12	0.1802	0.1774	1.1522	0.080*	
H4A	−0.026 (3)	0.317 (4)	0.9919 (19)	0.080*	
H4B	0.004 (3)	0.204 (4)	1.034 (2)	0.080*	
H1W	0.001 (2)	−0.214 (3)	0.7165 (14)	0.046 (10)*	
H2W	0.040 (3)	0.410 (5)	0.734 (3)	0.13 (2)*	

### Atomic displacement parameters (Å^2^)

**Table:**

	*U*^11^	*U*^22^	*U*^33^	*U*^12^	*U*^13^	*U*^23^
Zn1	0.0277 (2)	0.0478 (3)	0.0200 (2)	0.000	−0.00160 (16)	0.000
O1W	0.066 (2)	0.0431 (18)	0.0229 (16)	0.000	−0.0029 (15)	0.000
O2W	0.151 (5)	0.057 (3)	0.111 (4)	0.000	0.071 (4)	0.000
O1	0.0634 (15)	0.0664 (16)	0.0616 (16)	−0.0025 (13)	−0.0322 (13)	−0.0026 (13)
O2	0.0622 (15)	0.0615 (15)	0.0514 (14)	−0.0047 (12)	−0.0083 (12)	0.0277 (12)
O3	0.0537 (13)	0.0614 (15)	0.0383 (12)	−0.0128 (12)	0.0016 (10)	0.0083 (10)
N1	0.0270 (11)	0.0368 (13)	0.0253 (11)	0.0028 (9)	−0.0030 (9)	−0.0018 (9)
N2	0.0271 (11)	0.0375 (12)	0.0261 (11)	0.0024 (10)	−0.0054 (9)	−0.0002 (10)
N3	0.0267 (10)	0.0266 (11)	0.0215 (10)	0.0003 (9)	−0.0009 (8)	−0.0023 (8)
N4	0.0344 (12)	0.0418 (13)	0.0273 (12)	0.0038 (11)	0.0018 (10)	−0.0097 (10)
N5	0.0278 (11)	0.0394 (13)	0.0242 (11)	0.0010 (10)	−0.0028 (9)	0.0017 (9)
N6	0.0422 (13)	0.0328 (12)	0.0250 (11)	0.0006 (10)	−0.0047 (10)	0.0003 (9)
N7	0.0366 (13)	0.0393 (14)	0.0350 (13)	0.0044 (11)	−0.0049 (10)	0.0015 (11)
C1	0.0262 (12)	0.0264 (12)	0.0225 (12)	−0.0008 (10)	−0.0026 (10)	0.0001 (10)
C2	0.0278 (12)	0.0245 (12)	0.0275 (13)	0.0000 (10)	−0.0022 (10)	0.0012 (10)
C3	0.0326 (14)	0.0338 (14)	0.0380 (15)	0.0030 (12)	−0.0006 (12)	−0.0029 (12)
C4	0.0293 (14)	0.0428 (16)	0.0505 (18)	0.0073 (12)	−0.0015 (13)	−0.0043 (14)
C5	0.0302 (14)	0.0546 (19)	0.0430 (17)	0.0058 (13)	−0.0079 (13)	0.0075 (14)
C6	0.0300 (14)	0.0540 (18)	0.0288 (14)	0.0012 (13)	−0.0068 (11)	0.0038 (13)
C7	0.0278 (12)	0.0265 (12)	0.0261 (13)	0.0003 (10)	−0.0014 (10)	0.0010 (10)
C8	0.0328 (13)	0.0265 (13)	0.0252 (12)	−0.0028 (11)	−0.0027 (11)	0.0017 (10)
C9	0.0317 (13)	0.0338 (14)	0.0334 (14)	−0.0006 (12)	−0.0040 (11)	−0.0006 (12)
C10	0.0320 (14)	0.0390 (16)	0.0463 (17)	−0.0017 (12)	−0.0092 (13)	0.0050 (13)
C11	0.0438 (17)	0.0413 (16)	0.0384 (16)	−0.0059 (13)	−0.0173 (13)	0.0066 (13)
C12	0.0479 (17)	0.0404 (16)	0.0251 (13)	−0.0030 (13)	−0.0097 (12)	0.0019 (12)

### Geometric parameters (Å, °)

**Table:**

Zn1—O1W	2.008 (3)	N6—C12	1.346 (3)
Zn1—O2W	2.852 (5)	C1—C2	1.469 (3)
Zn1—N1	2.078 (2)	C2—C3	1.382 (4)
Zn1—N5	2.141 (2)	C3—C4	1.387 (4)
O1W—H1W	0.79 (3)	C3—H3	0.9300
O2W—H2W	0.92 (5)	C4—C5	1.386 (4)
O1—N7	1.243 (3)	C4—H4	0.9300
O2—N7	1.238 (3)	C5—C6	1.378 (4)
O3—N7	1.253 (3)	C5—H5	0.9300
N1—C1	1.319 (3)	C6—H6	0.9300
N1—N2	1.373 (3)	C7—C8	1.469 (3)
N2—C7	1.323 (3)	C8—C9	1.393 (4)
N3—C1	1.355 (3)	C9—C10	1.385 (4)
N3—C7	1.372 (3)	C9—H9	0.9300
N3—N4	1.426 (3)	C10—C11	1.377 (4)
N4—H4A	0.94 (4)	C10—H10	0.9300
N4—H4B	0.89 (4)	C11—C12	1.379 (4)
N5—C6	1.340 (3)	C11—H11	0.9300
N5—C2	1.358 (3)	C12—H12	0.9300
N6—C8	1.343 (3)		
			
O1W—Zn1—N1	97.28 (6)	N5—C2—C3	122.7 (2)
O1W—Zn1—N1^i^	97.28 (6)	N5—C2—C1	111.5 (2)
N1—Zn1—N1^i^	165.44 (12)	C3—C2—C1	125.9 (2)
O1W—Zn1—N5^i^	108.96 (6)	C2—C3—C4	118.4 (3)
N1—Zn1—N5^i^	97.73 (8)	C2—C3—H3	120.8
N1^i^—Zn1—N5^i^	77.47 (8)	C4—C3—H3	120.8
O1W—Zn1—N5	108.96 (6)	C5—C4—C3	119.3 (3)
N1—Zn1—N5	77.47 (8)	C5—C4—H4	120.3
N1^i^—Zn1—N5	97.73 (8)	C3—C4—H4	120.3
N5^i^—Zn1—N5	142.09 (12)	C6—C5—C4	118.9 (3)
O2W—Zn1—N1	82.73 (5)	C6—C5—H5	120.6
O2W—Zn1—N5	71.05 (5)	C4—C5—H5	120.6
Zn1—O1W—H1W	120 (2)	N5—C6—C5	122.9 (3)
C1—N1—N2	109.2 (2)	N5—C6—H6	118.6
C1—N1—Zn1	114.07 (16)	C5—C6—H6	118.6
N2—N1—Zn1	136.65 (16)	N2—C7—N3	109.8 (2)
C7—N2—N1	106.3 (2)	N2—C7—C8	123.3 (2)
C1—N3—C7	106.0 (2)	N3—C7—C8	126.9 (2)
C1—N3—N4	124.4 (2)	N6—C8—C9	123.1 (2)
C7—N3—N4	129.6 (2)	N6—C8—C7	116.7 (2)
N3—N4—H4A	105 (2)	C9—C8—C7	120.2 (2)
N3—N4—H4B	104 (2)	C10—C9—C8	118.1 (3)
H4A—N4—H4B	102 (3)	C10—C9—H9	120.9
C6—N5—C2	117.9 (2)	C8—C9—H9	120.9
C6—N5—Zn1	126.42 (18)	C11—C10—C9	119.4 (3)
C2—N5—Zn1	115.42 (16)	C11—C10—H10	120.3
C8—N6—C12	117.3 (2)	C9—C10—H10	120.3
O2—N7—O1	121.5 (3)	C10—C11—C12	118.8 (3)
O2—N7—O3	119.0 (2)	C10—C11—H11	120.6
O1—N7—O3	119.4 (2)	C12—C11—H11	120.6
N1—C1—N3	108.7 (2)	N6—C12—C11	123.2 (3)
N1—C1—C2	120.6 (2)	N6—C12—H12	118.4
N3—C1—C2	130.7 (2)	C11—C12—H12	118.4

Symmetry codes: (i) −*x*, *y*, −*z*+3/2.

### Hydrogen-bond geometry (Å, °)

**Table:**

*D*—H···*A*	*D*—H	H···*A*	*D*···*A*	*D*—H···*A*
N4—H4A···O2^ii^	0.94 (4)	2.25 (4)	3.100 (3)	149 (3)
N4—H4A···O2	0.94 (4)	2.55 (4)	3.192 (4)	126 (3)
N4—H4B···N6	0.89 (4)	2.13 (4)	2.875 (3)	141 (3)
N4—H4B···O3	0.89 (4)	2.43 (4)	3.005 (3)	122 (3)
O1W—H1W···O3^iii^	0.79 (3)	1.93 (3)	2.714 (3)	170 (3)
O2W—H2W···O1^iv^	0.92 (5)	2.02 (5)	2.900 (4)	161 (5)

Symmetry codes: (ii) −*x*, −*y*+1, −*z*+2; (iii) *x*, −*y*, *z*−1/2; (iv) *x*, −*y*+1, *z*−1/2.
